# Constructing national identity in public health crises: a comparative DHA study of China and the United States (2003–2023)

**DOI:** 10.3389/fpubh.2025.1688483

**Published:** 2025-12-15

**Authors:** Wei Wu

**Affiliations:** School of Marxism, Shanghai Jiao Tong University, Shanghai, China

**Keywords:** national identity, crisis discourse, public health, discourse historical approach, China, United States, UN general assembly

## Abstract

This study examines how China and the United States construct national identity in multilateral settings during public health crises through strategic discourse. Drawing on National Identity Theory and the Discourse Historical Approach (DHA), it analyzes speeches delivered at the United Nations General Assembly (UNGA) from 2003 to 2023, covering multiple crises including SARS, H1N1, Ebola, HIV/AIDS, and COVID-19. Through a longitudinal and cross-crisis comparative analysis, the study reveals evolving discursive patterns that reflect shifting self–other dynamics in global health governance. China consistently constructs an identity as a cooperative, responsible major power through inclusive and multilateral language, while the United States exhibits greater variation across administrations, often framing its identity through alliance-centered and leading position. By bridging discourse-historical analysis with corpus-based methods, this research offers one of the first systematic cross-crisis comparisons of identity construction in global health diplomacy. It highlights how crises serve as critical junctures for nations to redefine their international roles, providing insights into the communicative foundations of global health governance.

## 1 Introduction

Public crises test a nation’s governance capacity and its ability to communicate and shape national identity on the global stage. In such situations, national leaders often use public speeches to present policy positions, shape national images, and influence international perceptions. In highly visible multilateral arenas such as the United Nations General Assembly (UNGA), leaders must not only respond to the immediate challenges of a crisis but also clearly express national stances and seek international support. As Wodak (1) notes, political discourse reflects social and political contexts and is used by leaders to legitimize policies and shape collective identities internationally. These qualities enhance persuasive power and reveal how leaders construct their identities in cross-cultural and transnational communication. Recent studies have shown growing interest in how crisis discourse is constructed and transmitted, highlighting the central role of language in shaping public perceptions and national images (2, 3). Understanding how discourse strategies influence national identity during crises is therefore of both theoretical and practical importance.

China and the United States, as two of the most influential actors in the world, have attracted wide attention for their discursive interactions during public health crises. From the SARS outbreak (2003–2004) to the Ebola epidemic (2014–2015) and the COVID-19 pandemic (2020–2022), leaders from both countries have used the UNGA to present their crisis-response approaches. Political speeches in such contexts are both an exercise of power and an expression of identity. Leaders’ language choices in multilateral settings reveal their attitudes toward the international order and global governance. They also reflect how leaders understand their country’s role and self-identity. As domestic politics, international dynamics, and diplomatic goals change, leaders often adjust their linguistic stance and strategies. These adjustments provide important opportunities to study how states construct identity during crises. Based on this, the present study applies the Discourse-Historical Approach (DHA) to examine how Chinese and US leaders construct self-positioning and portray others during public health crises. National identity theory offers a strong framework for understanding the links between language, identity, relationships, and cognition.

**Figure 1:**
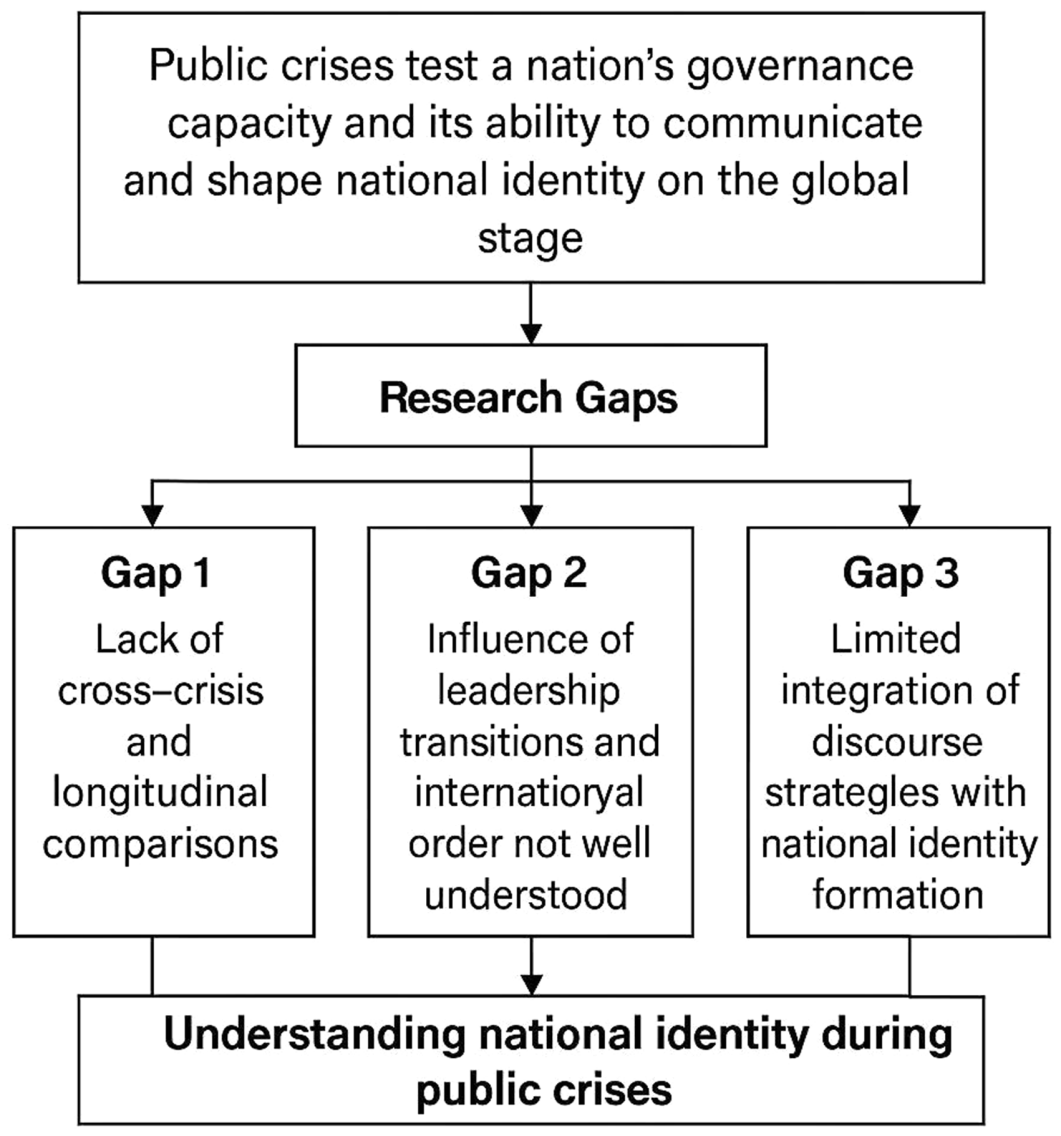
Research gaps schematic figure.

Although previous research has provided valuable empirical insights into the differences between China and US crisis discourse, several limitations remain. As a result, the internal logic between discourse strategies and identity construction has not been fully explained. This study summarizes the three major research limitations identified in previous studies and illustrates why addressing them is essential for advancing the understanding of national identity construction in crisis discourse (see Figure 1). Thus, this study seeks to address the following questions: What discourse strategies did China and US leaders adopt during major global public health crises from 2003 to 2023? How did these strategies contribute to the construction of national identity? How did they change across different crises and political stages, and what underlying logic of identity construction do these changes reveal?

To answer these important questions, this study conducts a longitudinal and cross-national comparison of how China and US leaders addressed public health crises in their twenty-year UNGA speeches, using corpus-assisted qualitative and quantitative methods. Covering the period from 2003 to 2023, this twenty-year timeframe includes several major global public health crises and spans key phases of political leadership transitions and shifts in the international order in both China and the United States. Thus, selecting speeches during this period allows the study to reveal both the stability and the dynamics of identity construction in their crisis discourses. This research draws upon national identity theory (4) as its main theoretical framework and combines it with the Discourse Historical Approach (DHA). National identity theory emphasizes that identity is a nation’s self-perception constructed through discourse in interaction with others, relatively stable yet adjustable in specific contexts. The DHA focuses on how discursive strategies (such as nomination, predication, and argumentation) operate within historical contexts and evolve over time. Combining these two approaches makes it possible to uncover how China and the US construct and reshape their identities through crisis discourse, and how these strategies change with the nature of crises, domestic politics, and shifts in the global order.

## 2 Literature review and theoretical framework

### 2.1 Crisis discourse and national identity

Discourse is central to the construction of national identity. Identity is not innate but socially constructed through interaction and discursive practices (4, 5). It emerges as both a product of discourse and a process that is continuously reshaped in interaction. As the vehicle of identity, language not only articulates national self-positioning but also delineates the boundary between “us” and “them,” serving as a key resource for identity strategies (6). National identity theory emphasizes that identity is discursively produced and reproduced in international exchanges, manifested in how a nation defines “who we are” and positions itself against the “other” (7, 8). From this perspective, examining self–other constructions provides critical insights into the mechanisms of identity formation and transformation.

Public health crises offer a particularly salient context for identity construction because of their visibility, transnational character, and political sensitivity (2). During crises, leaders must communicate policies while shaping domestic and international perceptions of the nation (3, 61). Crisis discourse thus performs a dual function: consolidating internal consensus to reinforce national identity and projecting a favorable image abroad (9). Leaders often adjust their narratives in response to diplomatic needs or domestic pressures (10). Common strategies include continuation, transformation, and exclusion, which can highlight distinctiveness or signal identity change (62). Keywords such as “we,” “nation,” “unity,” and “threat” frequently appear in political and media discourse, reinforcing cohesion and self–other distinctions. Yet, most studies emphasize the relative stability of identity and pay less attention to short-term, strategic adjustments triggered by crises.

In the field of global health diplomacy, national identity is continuously shaped and reshaped within the tension between globalism and nationalism. Zheng (11) argues that nationalism serves as a powerful force resisting globalization, especially the kind dominated by Western values. In contrast, Çınar (12) suggests that globalism, through the “global–local” dichotomy, reproduces the relationship between “us” and “them,” giving specific meaning to the construction of national identity. Among these debates, vaccine nationalism has become an important lens for understanding how national identity operates in times of global crisis. When a state treats vaccines as “mine” rather than “ours,” it reflects the failure of international cooperation and reinforces the core nationalist narrative of protecting one’s own citizens (13). Furthermore, discussions surrounding World Health Organization (WHO) reform, from the 2002–2003 SARS outbreak and the 2009–2010 H1N1 pandemic to the 2014–2016 Ebola epidemic and the COVID-19 pandemic (14–17), also reveal how nations construct and express their identities within global health governance. For instance, the United States often presents itself as a rule-maker and global leader (18), while China emphasizes its role as a provider and collaborator in advancing global health (19).

In this study, “national identity” and “national image” are treated as related but distinct concepts. National identity refers to how a nation defines itself through discourse, history, and diplomacy. It is relatively stable but contextually adaptable (4, 6). National image, in contrast, concerns how external audiences perceive and evaluate a nation (20, 21). These two dimensions intersect in crises: leaders’ discourse can strengthen internal identification while simultaneously influencing external perceptions (9, 22). However, their emphases differ: image addresses “how others see us,” whereas identity focuses on “how we define ourselves.” This study centers on national identity construction, while also considering the implications of crisis discourse for national image, thereby offering a more comprehensive understanding of Chinese and US strategies.

In summary, national identity theory provides a strong foundation for analyzing crisis discourse. It highlights how identities are socially constructed and discursively reproduced, and it clarifies how leaders reinforce unity and positioning through self–other distinctions. It also helps distinguish identity from image, allowing us to examine the dual functions of crisis discourse in domestic cohesion and international communication. Within the sensitive context of global public health crises, this framework enables a comparative analysis of how Chinese and American leaders construct “who we are” and define the “other.”

### 2.2 Discursive practice of China and the United States in public health crises

In the US context, extensive research has examined the discursive practices of political leaders during the COVID-19 pandemic. Trump’s speeches, in particular, have received considerable scholarly attention, with analyses focusing on how he employed crisis discourse, pragmatic strategies, and communicative resources to shape pandemic narratives and engage the public (23). Other studies contrasted Trump’s passive responses with the proactive communication strategies of several governors, highlighting divergences between federal and state-level crisis discourse (24, 25). Research on Biden’s pandemic rhetoric constitutes a deliberate discursive construct, deploying a range of linguistic features and strategies to advance specific communicative and ideological aims (26–29). Scholars have further identified Biden’s frequent use of metaphors such as “unity,” “object,” “person,” “space,” and “war” (30), p. 810, along with his reliance on positive self-presentation strategies to construct a leadership image (31).

In contrast, China expanded its global influence during the pandemic by providing medical supplies and expertise (32). Scholars generally view COVID-19 as a critical turning point in China’s international communication and national identity reconstruction (33), with profound implications for the US-led international order (32, 34). Much of the research employs critical discourse analysis and metaphor analysis to decode Chinese official and diplomatic discourse. For instance, Yang and Chen (10) demonstrate that Chinese official narratives intertwined elements of globalism and nationalism to strengthen the image of China as a “responsible major power.” Similarly, Yu (35) shows that China Daily used metaphorical narratives to frame the pandemic as a “global battle for a shared future.” Further studies of the “China Keywords” online platform (36) and the Ministry of Foreign Affairs’ official statements (37) reveal China’s consistent emphasis on solidarity and cooperation in pandemic discourse.

Before the outbreak of COVID-19, both China and the United States had accumulated extensive experience in discourse practice during previous public health crises. In the United States, relevant studies primarily focused on the interaction among governmental agencies, the media, and the public. For instance, (38) examined the information disclosure mechanisms employed by the Centers for Disease Control and Prevention (CDC) during crises and their impact on public trust. Sell et al. (39) investigated the dissemination of misinformation by the US government and media during the Ebola outbreak. Gesser-Edelsburg et al. (40) analyzed how the Obama administration’s linguistic framing of Ebola shaped social action and policy decision-making. Furthermore, Herek (41) conducted an in-depth analysis of the stigmatizing discourse surrounding HIV/AIDS, revealing the role of language in shaping social prejudice and public perceptions. In China, during the 2003 SARS crisis, national leaders took decisive actions to dispel misinformation, regulate public emotions, and restore social order (42). Drawing on critical metaphor analysis (63), Chiang and Duann (43) revealed how the metaphor of “disease as war” constructed national narratives of “self” and “other” within SARS-related discourse. In addition, (44), through an analysis of Chinese official media coverage of the Ebola outbreak in Africa, found that Chinese media tended to highlight the achievements, bravery, and humanitarian efforts of African people—an approach that stands in sharp contrast to the more negative or crisis-centered framing often seen in Western media reports.

Comparative research across crises and between China and the United States remains limited. Zhang (45) conducted a framing analysis of China Daily’s coverage of SARS and COVID-19, though based on a small corpus. At the bilateral level, Wang (46) compared Xi Jinping’s and Trump’s speeches to examine legitimization strategies in political rhetoric, while Al-Saaidi (47) analyzed official statements from both governments in March 2020, highlighting ideological divergences in Sino-US diplomatic discourse during the pandemic.

Research has shown that the discourse of governments and the media in public health crises serves not only to communicate information but also to shape national identity. However, most research focuses on single crises (e.g., SARS or COVID-19) or short-term contexts, with limited systematic comparison across crises and time periods. There is also insufficient examination of how discursive strategies evolve within the same country under different leaderships or international circumstances. Furthermore, the internal logic of identity construction during crises remains fragmented, lacking a coherent comparative framework. Finally, questions regarding the influence of regime change and domestic political systems on identity strategies remain underexplored in empirical cross-national research (21, 22).

This study aims to analyze the discursive strategies employed by China and the United States in public health crises through the lens of national identity theory. Adopting a corpus-assisted historical discourse analysis, the empirical section employs a longitudinal comparative approach to systematically trace the evolution of Chinese and American public health crisis discourses from 2003 to 2022, examining how these discursive practices interact with and contribute to the construction of national identity. Previous applications of national identity theory in diverse contexts such as crisis leadership (48), US presidential campaign rhetoric (49), and China’s crisis communication (50) have demonstrated the theory’s analytical strength in explaining how discourse shapes collective identity and national self-image. Building on these insights, the present study integrates national identity theory directly into the analysis of public health crisis discourse, thereby bridging the existing gap between discursive practices and identity construction in cross-national and longitudinal crisis contexts.

## 3 Methodology

### 3.1 Discourse-historical approach

The Discourse-Historical Approach (DHA) integrates macro-level contextual analysis with micro-level linguistic examination, providing a systematic framework for the study of political discourse. As a key branch of Critical Discourse Analysis, DHA pays particular attention to the ways in which discursive practices change over time and contribute to the construction of national identity (6, 51). From a cognitive perspective, Wodak conceptualizes discourse as a dynamic, context-dependent semiotic practice embedded in social action. DHA also emphasizes the intertextual relationship between texts, genres, and discourses, while highlighting the influence of historical, social, and institutional factors in shaping linguistic production (64). Its historical dimension underscores two key principles: (1) situating discourse events within their historical backgrounds and origins wherever possible, and (2) tracing their trajectories of change over time (6).

At the operational level, DHA identifies five core discursive strategies: nomination strategies, predication strategies, argumentation strategies, perspectivization strategies, and intensification/mitigation strategies (1). These strategies can be selectively employed, adapted, and combined in different political contexts to achieve identity construction and political objectives. In line with the research focus, this study concentrates on nomination, predication, and argumentation strategies, which are summarized in Table 1.

**Table 1:** Discursive strategies in DHA.

Discursive strategy	Definition and function	Linguistic realization	Role in identity construction
Referential strategies	Identifying and classifying persons, groups, events, or phenomena through naming, reference, and labeling	Names, categorical labels, collective terms, metaphorical references	Establish boundaries between “self” and “other,” defining group identity
Predication strategies	Attributing specific qualities or characteristics to the referent	Adjectives, verbs, nominalizations, comparative structures, metaphors	Reinforce or weaken particular identity traits, shaping positive/negative images
Argumentation strategies	Providing justification and legitimation for positions or actions through topoi and reasoning rules	Reasoning patterns such as the topos of usefulness, responsibility, or justice	Legitimize self-identity claims or rationalize othering

The advantages of DHA for the present study are threefold. First, its diachronic orientation aligns well with the research design. By analyzing discourse in its historical context and tracing its transformations, DHA enables the study to uncover both continuities and shifts in discursive strategies across different public health crises from 2003 to 2023. Second, its sensitivity to context enhances explanatory power in cross-national comparison. By embedding discourse in its social, political, and institutional settings, DHA illuminates the deeper reasons behind divergent discursive patterns in China and the United States under similar crisis conditions, thus avoiding the limitations of “decontextualized” analysis. Third, its multidimensional strategic framework provides methodological tools to systematically identify and compare how Chinese and US leaders construct national identity through crisis discourse. Previous studies demonstrate that DHA has been widely applied in the analysis of election speeches, immigration policies, and international diplomacy (1, 52, 53), proving its strength in revealing the discursive construction of group identity.

Complementing DHA, national identity theory (4) provides the conceptual and interpretive framework for this research. The theory emphasizes that national identity is continuously produced and reproduced through interactions with the “Other,” as well as through narrative and symbolic practices (4, 7). Combining DHA with national identity theory allows this study to operate at two levels: analytically, DHA identifies the concrete strategies and linguistic features of crisis discourse; interpretively, identity theory explains how these strategies serve to define the Self and delineate the other. This methodological-theoretical complementarity enables the research not only to describe the formal characteristics of linguistic strategy, but also to reveal their underlying political logic and significance in international relations.

### 3.2 Corpus

Corpus linguistics provides an effective set of tools for the systematic analysis of large-scale textual data. Its core methods include automated retrieval and statistical comparison of keywords across corpora, as well as the generation of word lists, collocation analysis, and the identification of co-occurrence patterns (65). Keywords, word frequency, and semantic collocation constitute the basic components of corpus linguistics, enabling researchers to uncover latent linguistic patterns and traces of ideology in extensive datasets. This approach has been widely applied in studies of political and media discourse. For instance, examined online NHS health communication to reveal underlying linguistic tendencies in patient feedback, while employed corpus methods to analyze national image construction in Xinhua News texts. In this study, corpus linguistics functions as a quantitative tool to identify high-frequency topics and keyword patterns in the speeches of Chinese and US leaders at the United Nations General Assembly (UNGA), thereby laying the groundwork for subsequent qualitative interpretation.

### 3.3 Data selection

This study selects the general debate speeches of China and the United States at the UNGA as the research corpus, primarily due to the United Nations’ unique position in international political communication. The UNGA General Debate, held annually, is regarded as a critical diplomatic stage. For member states, it serves as a platform where national leaders present an idealized self-identity to the international community (66), while also influencing how their country is perceived and evaluated abroad through public oratory (67). As a strategic act of communication, leaders’ speeches at the UNGA convey not only policy preferences and national positions, but also reflect broader diplomatic agendas and international orientations China and the United States—as two of the world’s most influential powers—use their UNGA statements on public health crises not only to signal foreign policy priorities but also to actively participate in shaping international perceptions of their national identities. Accordingly, the UNGA General Debate offers an ideal data source for analyzing identity-construction strategies during public health crises.

In selecting the data, this study considered both the representativeness and the limitations of the corpus. The institutional characteristics of the United Nations General Assembly (UNGA) General Debate further enhance its representativeness. On the one hand, the speeches are highly autonomous and non-negotiated (68), providing governments with a relatively free space to express their policy positions, diplomatic goals, and views on international issues. On the other hand, the debates cover the most significant global topics of each year (69), which allows for comparison of different countries’ crisis discourses within a shared communicative context. Thus, these features make the UNGA General Debate an ideal setting for cross-national comparisons of identity-construction strategies.

The corpus used in this study mainly represent national expressions in an international diplomatic context rather than in domestic political settings. Leaders tend to use different rhetorical strategies in these two contexts: domestic speeches or media statements often emphasize internal political mobilization, whereas international addresses focus more on diplomatic image and global responsibility. As this study concentrates on national identity construction in the context of global public-health governance, the UNGA discourse is considered both representative and analytically valuable. Although this selection may introduce some bias, using a consistent international forum ensures comparability and focus on diplomatic identity construction. This study also recognizes certain limitations in corpus scope selection. Nevertheless, future research will expand the data scope to include other United Nations platforms (e.g., the World Health Assembly, UN Security Council) and selected domestic policy statements to provide a more comprehensive account of national identity discourses.

### 3.4 Data collection

For corpus construction, this study employs KH Coder as the primary analytical tool and builds two small-scale parallel corpora to examine how China and the United States adapted their communicative strategies in public health crises and how such strategies contributed to national identity construction. The data was downloaded from the United Nations General Debate Corpus (UN-GDC) available via Harvard Dataverse (DOI: 10.7910/DVN/0TJX8Y). The temporal scope of analysis spans from 2003 to 2023, covering multiple global public health crises of major significance: the 2003 SARS outbreak, the 2009 H1N1 influenza pandemic, the 2014 Ebola epidemic in West Africa, the COVID-19 pandemic from 2020 onwards, as well as the long-term global challenge of HIV/AIDS. The starting year (2003) was chosen because the World Health Organization (WHO) declared SARS a global epidemic that year, while the endpoint (2023) marks the WHO’s announcement that COVID-19 no longer constituted a Public Health Emergency of International Concern (PHEIC). This time frame thus captures a full twenty-year cycle of public health crises and the accompanying shifts in discursive strategies.

To ensure completeness and comparability, the dataset covers all speeches delivered by Chinese and US leaders from 2003 to 2023, including those by heads of state, heads of government, and in exceptional cases, foreign ministers or other senior officials. After determining the crisis events, time periods, and speaker lists, speeches were systematically examined for passages referencing public health crises. All speech texts were retained in their original languages without translation to preserve the authenticity of linguistic features for analysis.

For this study, a “health crisis” is defined as any issue concerning major epidemics, infectious disease outbreaks, or global/regional public health threats and their responses. The screening process proceeded as follows: first, each speech was read in full, supplemented by keyword searches (e.g., epidemic, pandemic, public health, infectious disease, COVID, vaccine) to identify candidate passages. Second, manual coding by the research team was conducted to retain only the sections explicitly related to health crises, while excluding general health references unrelated to crisis contexts. Detailed selection criteria are provided in Table 2.

**Table 2:** Selection criteria for public health crisis corpus.

Category	Selection content
Disease names	SARS, COVID-19, AIDS, HIV, Ebola, H1N1, H7N9, Influenza
Health	WHO, health, public health, epidemic, pandemic, virus, infection, disease
Crisis	Crisis, outbreak, prevention, treatment, vaccination, medical aid
Filtering principles	Keyword search and manual judgment

In total, 42 original UNGA speeches by Chinese and US leaders from 2003 to 2023 were collected, comprising 124,856 words (see Table 3). The study identified and highlighted text segments containing predefined screening keywords. These segments could consist of one or more sentences, or even entire paragraphs, but were included only if they conveyed relevant and explicit meanings related to health crises. For systematic analysis, only the health-crisis-related passages within these speeches were subjected to both quantitative and qualitative examination (see Table 2). Passages unrelated to health crises, while excluded from the core analysis, were preserved as background material to contextualize national policy orientations and the relative salience of health issues in broader diplomatic agendas. All texts were converted into plain TXT format and labeled with identifiers combining “year + crisis event + country + speaker” to facilitate subsequent retrieval and analysis.

**Table 3:** Corpus sources and scale.

Category	Description
Sources	The United Nations General Debate Corpus (UN-GDC) available via Harvard Dataverse (DOI: 10.7910/DVN/0TJX8Y)
Time span	2003–2023
Sample selection	Annual speeches by Chinese and US leaders, totaling 42 speeches
Analysis scope	Paragraphs related to public health crises used as the analytical corpus
Word count	Total original corpus: 124,856 words (US: 79,081 words; China: 45,775 words). Total word count after filtering: 7,426 words.

### 3.5 Data analysis procedure

This study applies a systematic, multi-step analytical procedure to examine the 42 UNGA speeches (2003–2023) delivered by Chinese and US leaders on public health crises, using corpus-based methods in conjunction with the Discourse-Historical Approach (DHA). A triangulated approach integrating quantitative and qualitative analyses enhances the empirical grounding and analytical coherence of this study.

Step 1: Quantitative corpus analysis.

Prior to the main analysis, all 42 speeches were preprocessed. First, the raw texts were cleaned by removing punctuation marks, numbers, and special characters. Second, common stop words were excluded using the publicly available English stop word list from NLTK, in order to eliminate grammatical function words and filter out certain high-frequency terms that were irrelevant to the research topic. Finally, lexical normalization was performed using the Porter stemming algorithm to reduce morphological variations and enhance analytical consistency.

The first stage involved corpus-assisted quantitative analysis using KH Coder. Following (70), keywords and high-frequency lexical items are considered not only indicators of underlying ideological orientations and strategic preferences but also the foundation for subsequent collocation and co-occurrence analysis. Under identical statistical parameters and stop-word settings, high-frequency nouns were extracted separately from the China and US corpora. These wordlists served as the basis for identifying central issues and core concepts across different crisis contexts. Comparative keyword lists and frequency distributions were then analyzed to detect both thematic differences and commonalities in the two countries’ crisis discourses. The findings from this stage were later integrated with qualitative interpretation to uncover the identity-construction patterns underlying these discursive variations.

Step 2: Co-occurrence and semantic network analysis.

Building upon the high-frequency word analysis, the study conducted co-occurrence analysis to reveal semantic associations and co-textual patterns among the core vocabulary in each corpus. Co-occurrence mapping not only reflects the thematic organization and strategic lexical pairings of crisis discourse but also highlights how leaders construct self-identity and portray the “Other” through specific word combinations. In this study, the strength of co-occurrence relations was measured using the Cosine coefficient in KH Coder (Version 3.0). Kruskal’s non-metric multidimensional scaling (MDS) was employed to project words into a three-dimensional semantic space to visualize the spatial relationships among discourse themes. The stress values were 0.192 for the Chinese corpus and 0.185 for the US corpus, indicating good model fit (see Table 4).

**Table 4:** Analytical tools, parameter settings, and robustness checks.

Analysis type	Tool and version	Parameter settings	Robustness check
Keyword extraction	KH Coder 3.0	Minimum frequency ≥ 5; keywords selected by term frequency statistics	Adjusted word segmentation methods; validated the stability of keyword lists, network structures, and community divisions through checks on co-occurrence size and centrality values.
MDS co-occurrence analysis	KH Coder 3.0	Kruskal method; Cosine distance; 3 dimensions; stress value (China = 0.192, US = 0.185); window span ±5 words	
Collocation analysis	KH Coder 3.0	Node words set as China/United States; window span ±5 words	
Strategy coding	Manual coding	Two researchers independently coded according to the DHA framework	Randomly sampled health crisis-related paragraphs; two researchers independently applied the three main DHA strategies; compared original coding results; Cohen’s K = 0.85, indicating good inter-coder reliability.

Step 3: Qualitative discourse-historical analysis.

Following the quantitative phase, DHA was employed for in-depth qualitative analysis, allowing for a nuanced understanding of the strategies through which national identities were constructed during different health crises. In line with Lakoff’s (54) principle of prototypical sampling, the study adopted a “selective reduction” strategy (55) to select representative passages from different crisis stages. This ensured that the analysis covered both core discourse and less conventional textual elements. The selected texts were line-by-line coded to identify the application of nomination, predication, and argumentation strategies. The results of the co-occurrence analysis were then integrated with close reading of the original speech passages, enabling the identification of both continuity and shifts in discursive strategies across crises.

All analytical tools, parameter settings, and robustness checks are detailed in Table 4 to ensure transparency and replicability of the research process.

## 4 Results

### 4.1 Corpus analysis

#### 4.1.1 Keywords

**Figure 2:**
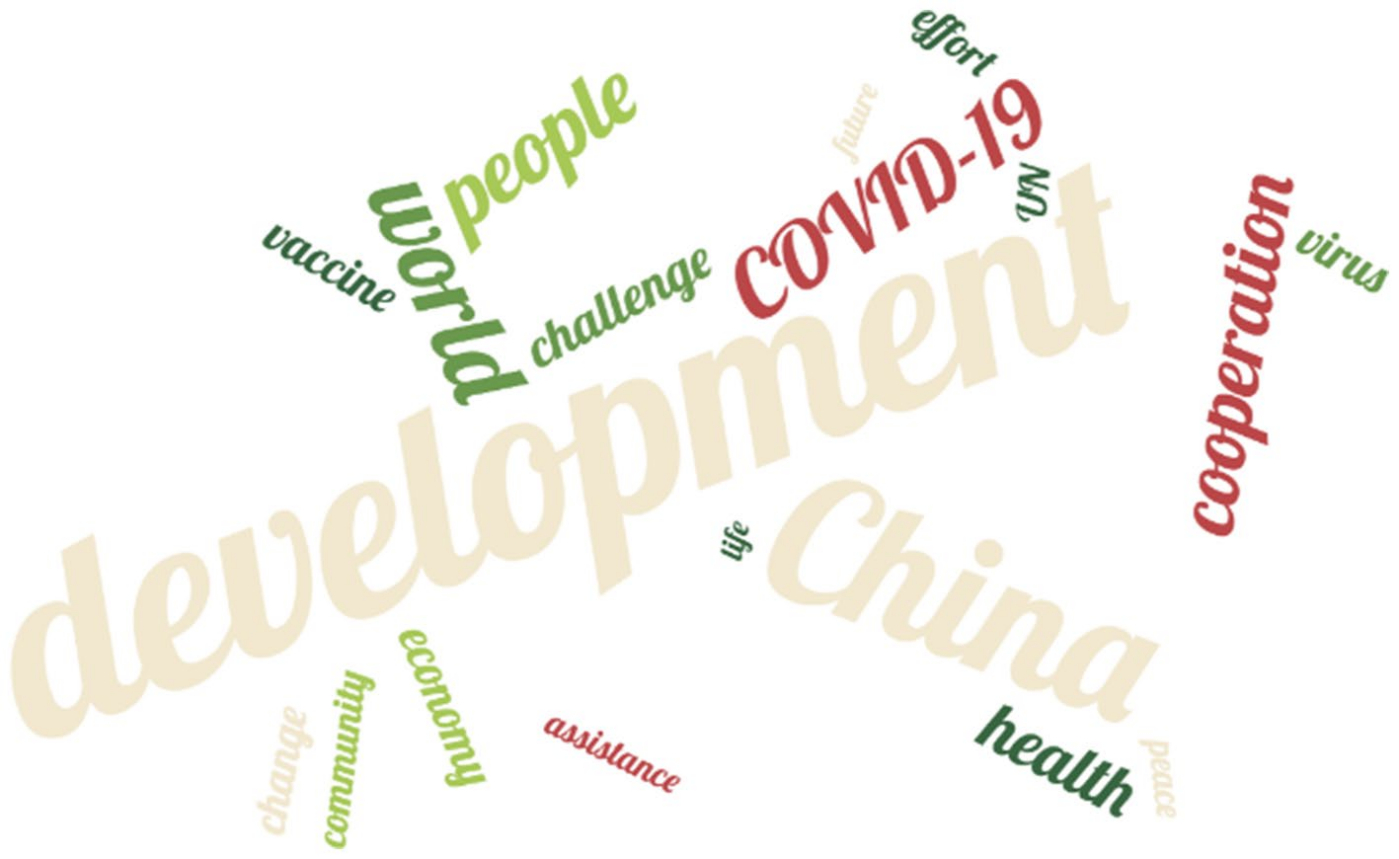
Word cloud of high-frequency nouns (China).

**Figure 3:**
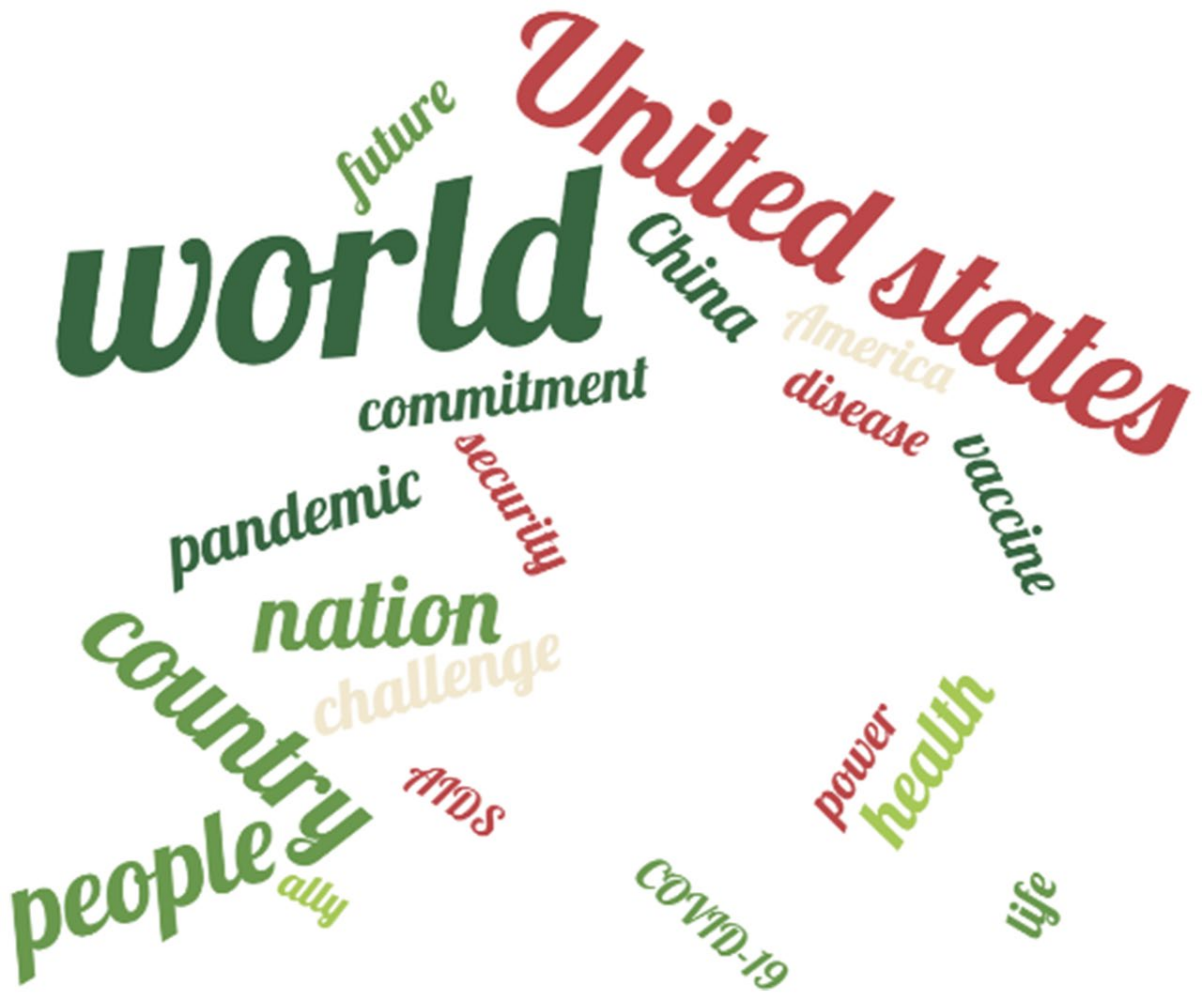
Word cloud of high-frequency nouns (the US).

This section presents the corpus analysis of the Chinese and US datasets in two parts: keyword analysis and co-occurrence analysis. Following Paul Baker’s (56) suggestion, keyword lists from different corpora were compared to identify statistically significant keywords that occur more frequently in one corpus than in the other. Figures 2, 3 displays the word cloud of top 20 high-frequency nouns, with results for Chinese leaders on the left and for US leaders on the right.

The keyword analysis reveals four core thematic clusters in the Chinese leaders’ corpus. First, high-frequency terms such as development (55), country (51), cooperation (18), and assistance (8) form a “development, cooperation and win–win” narrative frame. Public health crises are situated within a broader “global common development” narrative, emphasizing the need to address public health gaps by enhancing the capacity of developing countries. Second, vaccine (11) and COVID-19 (20) co-occur alongside cooperation (18) and assistance (8), reflecting China’s positioning of vaccines as a global public good and its emphasis on improving equity and accessibility through multilateral cooperation and external aid. Third, the co-occurrence of China (39), UN (9), and community (9) highlights China’s image as a major developing country within the multilateral system, stressing the responsibilities it assumes as a member of the “international community.” Finally, economy (11), change (10), and future (7) point to long-term issues such as economic restructuring and climate change, underscoring China’s framing of public health crises as manifestations of developmental imbalances and echoing its policy calls for long-term structural governance. These thematic findings are logically consistent with the subsequent analysis of discursive strategies.

The US leaders’ corpus likewise exhibits four prominent themes. First, world (32), challenge (9), security (7), and America (7) together form a “world, challenge and security” discourse chain, framing public health crises as external shocks to “the security of America and its allies,” and emphasizing the preservation of the US-led international order and strategic security. Second, while vaccine (8) and COVID-19 (6) also appear in the US corpus, their close association with ally (6) and commitment (8) indicates a strong alliance-based orientation in vaccine distribution, treating vaccines and medical resources as strategic tools and prioritizing the needs of allies and partners. Third, the combination of United States (20), nation (13), and power (7) underscores the link between national identity and hard power, while the pairing of America (7) with future (8) highlights US leadership within the alliance system and its strategic vision. Finally, life (7), disease (7), and AIDS (6) indicate urgent responses to specific diseases, while commitment (8) and future (8) reflect a pattern of “immediate pledges–shaping the future” characteristic of short-term political declarations in times of crisis.

In sum, the keyword analysis reveals the core concerns and emphases of Chinese and US leaders in the context of public health crises. China’s keyword network centers on “development–cooperation–multilateralism,” stressing long-term structural governance and the notion of global public goods; the United States, by contrast, orients around “security–alliances–leadership,” emphasizing strategic security and the maintenance of the international order. These differences not only reflect the two states’ policy priorities and identity narratives under crisis conditions, but also provide quantitative evidence for interpreting their discursive strategies. Building on this, the next step word co-occurrence analysis will examine how these keywords are combined in the discourse and how they are semantically associated, thus providing a more detailed account of the linguistic pathways through which identity construction occurs across crises.

#### 4.1.2 Word co-occurrence

This section applies KH Coder to conduct a co-occurrence network analysis of the portions of the 2003–2023 corpus relating to “public health crises.” Following, the analysis combines keyword methods with semantic domain approaches, enabling macro and micro-level examination to uncover detailed linguistic features.

**Figure 4:**
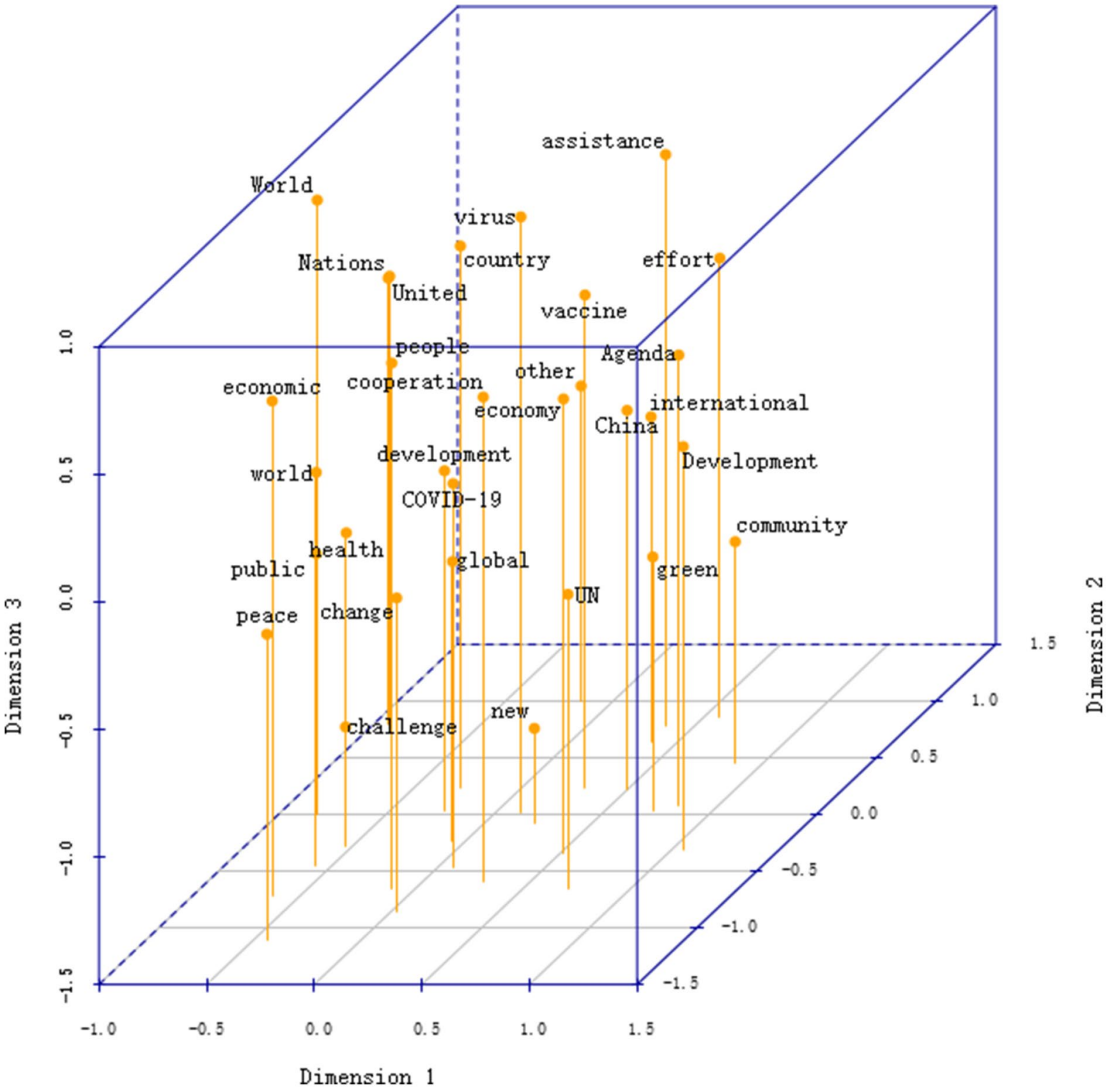
Three-dimensional MDS map of the Chinese corpus.

In the three-dimensional semantic space shown in Figure 4 (stress = 0.192), four interrelated semantic domains structure China’s crisis discourse. Along Dimension 1, the negative pole clusters a global governance semantic field (world, organization, united, international, governance, support), reflecting the official framing of health crises as matters of global public health governance. In contrast, the positive pole features a national development semantic field (China, development, community, share, win–win), which recontextualizes the same crisis within a China–Global South common development narrative that emphasizes South–South cooperation and shared progress. Dimension 2 differentiates pragmatic action from normative aspiration. The lower area contains terms related to immediate and concrete responses (virus, assistance, vaccine), while the upper region includes more abstract and forward-looking expressions (peace, green, community, change). This vertical progression illustrates the discourse’s movement from short-term crisis management toward long-term visions of global health and sustainability. Dimension 3 captures a strategic and temporal contrast between challenge-oriented terms (crisis, effort, challenge) and transformative ones (new, global, development), suggesting a rhetorical shift from reactive defense to proactive global engagement. These three dimensions converge around several bridging nodes, “COVID-19, people, global, development,” which connect the clusters of pandemic management, multilateral cooperation, and the “community of shared future for humankind.” Together, they depict an integrative semantic configuration that situates China’s health crisis discourse within a broader narrative of cooperation, development, and shared destiny.

**Figure 5:**
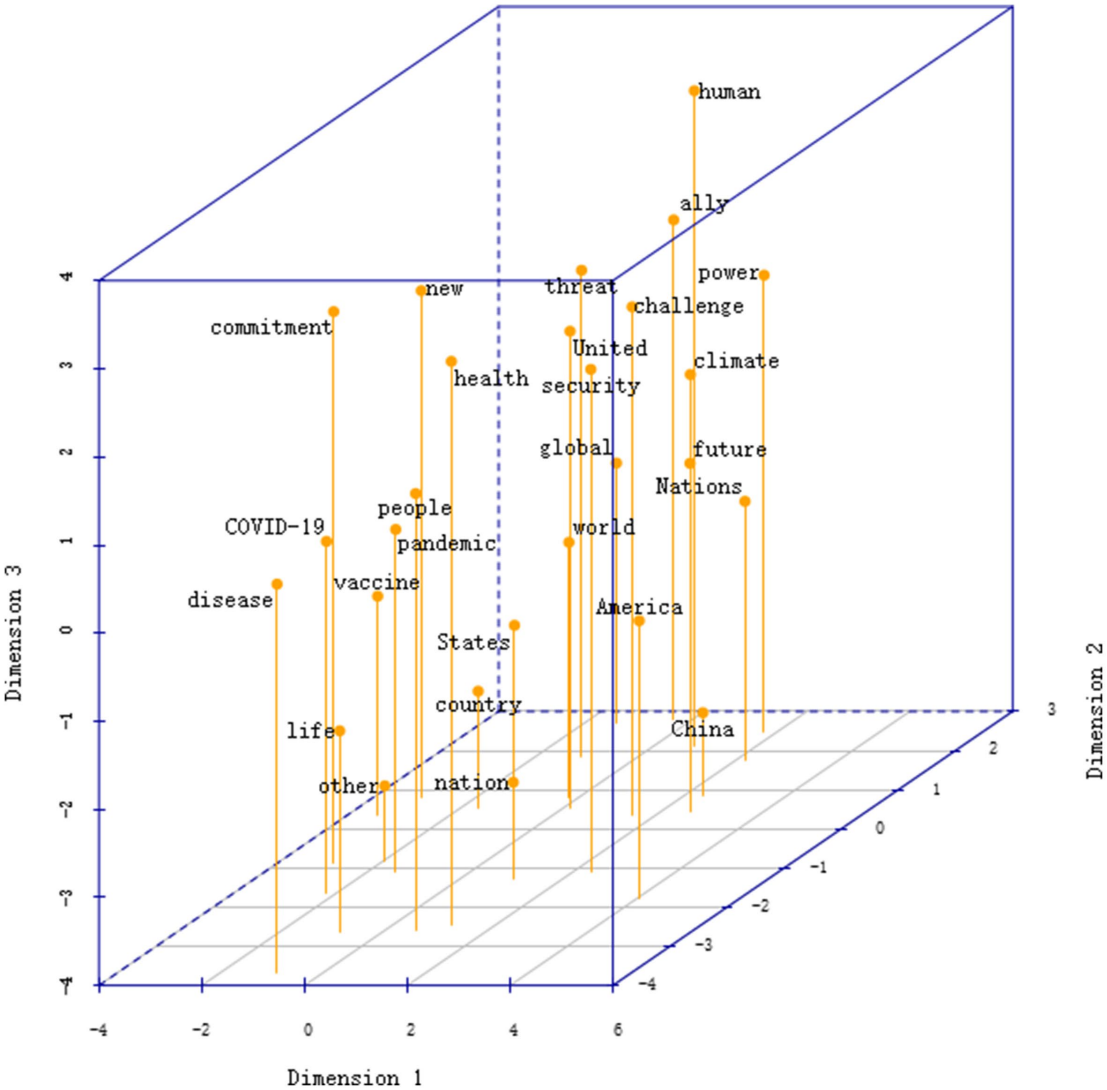
Three-dimensional MDS map of the US corpus. Node size is proportional to word frequency in the underlying data; all nodes are displayed at uniform size in the 3D projection for clarity. Spatial distance represents semantic similarity based on co-occurrence strength. The configuration illustrates the overall semantic structure of the China and US crisis discourse. Coordinate signs indicate statistical orientation only and carry no evaluative meaning.

In contrast, the US MDS configuration in Figure 5 (stress = 0.185) reveals a more differentiated and hierarchical semantic structure. Along Dimension 1, the distribution of America, United States, ally, partner, and commitment delineates a narrative of “revitalized US global leadership,” emphasizing alliance solidarity and international responsibility. At the opposite end, words such as China, power, threat, and security cluster together, framing health crises as “great-power competition and systemic challenges.” Dimension 2 introduces a security–value axis, linking threat, climate, power, and future, which connects health and climate agendas within a shared discourse of global risk management. Dimension 3 captures a humanitarian–strategic gradient, ranging from disease, life, and vaccine at the lower end to human and ally at the top, reflecting the discursive transition from domestic crisis response to moral and strategic legitimation. In US discourse, vaccine is positioned as a symbolic resource in alliance politics, with priority given to safeguarding allied interests, while security sits at the intersection of great-power competition and climate security, linking health crises to broader geopolitical and supply chain security concerns.

The discourse networks of China and the United States present two structurally symmetrical but semantically divergent narrative axes: China’s extends from global governance to development assistance, framing national interests within the legitimacy of multilateralism; the United States’ extends from alliance leadership to great-power rivalry, framing security threats within a value-based alliance system. Both employ vaccine and security as cross-cluster bridging symbols, yet China links security to biosafety and a “community of shared future for humankind,” whereas the United States associates it with geopolitics and supply chain security. This divergence reflects fundamental differences in how the two states construct identity and position themselves internationally during public health crises. In sum, China’s discourse exhibits an integrative and cohesive configuration centered on development, cooperation, community, and international, indicating an integrative discourse orientation, whereas the US discourse presents a segmented and hierarchical structure on public health, security, and leadership, indicating a differentiated discourse orientation.

### 4.2 Analysis of discursive strategies

The preceding section identified the keywords and thematic distributions in Chinese and US public health crisis discourse, laying the foundation for understanding the identity construction mechanisms behind these themes. This section turns to the process of discourse production, focusing on how these themes are shaped through specific discursive strategies. As Wodak (6) has noted, discourse production and dissemination are not random but rely on a series of relatively precise and purposeful action plans, including speech acts, designed to achieve particular social, political, psychological, or linguistic objectives. Drawing on the Discourse Historical Approach (DHA), this study concentrates on three core strategies highly relevant to identity construction: naming strategies, predication strategies, and argumentation strategies.

#### 4.2.1 Naming strategies

Naming strategies concern the ways in which political discourse identifies social actors, events, or phenomena through the use of proper nouns, titles, referential expressions, and other forms of designation, thereby expressing attitudes, stances, and value orientations (71). In the context of health crises, naming strategies reveal how speakers position both “self” and “other,” and thus reflect a state’s path of identity construction on the international stage.

An index line analysis of nominal references to “China/Chinese” and “United States/American” shows significant differences in the naming strategies employed by China and the United States in crisis discourse. China tends to use inclusive and cooperative designations, emphasizing friendship, assistance, and international cooperation to project the identity of a responsible, win–win partner. For Excerpt, in responding to the Ebola outbreak (Excerpt 1), China employed emotive reference by calling Africa a “good brother” and stressing that it would “stand firmly with the African people” during the crisis—thus underscoring its image as a South–South cooperation partner. During the COVID-19 pandemic (Excerpts 2, 3), China rejected Western stigmatization, framed vaccines as a global public good, and advocated for international cooperation under the WHO framework, thereby reinforcing its role as a “pioneer in global anti-pandemic efforts” and “provider of public goods.”

*Excerpt* 1: As a good brother and partner of Africa, with whom it shares the good and the bad, China will continue to stand firmly with the African people and to support and assist them to the best of its ability.

*Excerpt* 2: We should follow the guidance of science, give full play to the leading role of the World Health Organization, and launch a joint international response to beat this pandemic.

*Excerpt* 3: Any attempt of politicizing the issue or stigmatization must be rejected.

In contrast, the United States is more inclined toward bloc-based designations (e.g., “our allies”), using such references to strengthen its international claims and construct an active self-image of leadership. Notably, after the Biden administration took office, US naming strategies shifted to place greater emphasis on cooperation with partners and allies, in an effort to win international support for the new administration (Excerpt 4).

*Excerpt* 4: And I am in this Hall today to share with the General Assembly of the United Nations how the United States intends to work with partners and allies to answer these questions and the commitment of my new Administration to helping to lead the world toward a more peaceful, prosperous future for all people.

In sum, the naming strategies adopted by China and the United States during public health crises reflect their respective self-positioning in the international order. China’s inclusive and cooperative designations situate health crises within the framework of South–South cooperation and the provision of global public goods, reinforcing its image as a responsible major power. The United States, by contrast, relies more on bloc-based references, embedding crises within alliance politics and security narratives to consolidate its leadership identity. It is noteworthy that US naming strategies softened somewhat following the change of administration, demonstrating the flexibility of such strategies in response to political shifts. This strategic divergence provides important context for the subsequent analysis of predication and argumentation strategies.

#### 4.2.2 Predication strategies

Within DHA, predication strategies operate through the use of evaluative adjectives—either positive or negative—in the predicate components of discourse to assign specific qualities to social actors, objects, or phenomena (57). We conducted a collocation search for adjectives in the Chinese and US corpora, with the most frequently occurring adjectives shown in Table 5.

In Chinese UN General Assembly speeches concerning public health crises, predication strategies predominantly center on positively evaluative adjectives, especially in narratives of self-presentation and cooperative engagement. As shown in Table 5, when describing its own public health capacities, China frequently employs economically oriented adjectives that convey scale and quality (e.g., economic, high-quality, largest), highlighting advantages in key areas such as medical supply production capacity and diagnostic capability. For instance, in Excerpt 5, largest and high-quality simultaneously emphasize scale and service quality, while economic underscores accessibility and cost-effectiveness.

**Table 5:** Key adjective collocates associated with the identity labels “China, the United States” based on KH coder’s KWIC concordance analysis.

China	The United States
Economic, international, shared, high, historic, positive, ready, all-out, beautiful, high-quality, largest, entire, firm, first, heartfelt, heroic	Many, military, other, more, central, enduring exceptional, last, ready, human, essential, historic, ongoing, immediate, national

These adjective collocates are selected based on KH coder’s KWIC concordance analysis which uses the scale of Mutual Information to indicate the collocation between the node words (i.e., identity labels) and their adjective collocates.

*Excerpt* 5: China has already sent emergency aid supplies to the Democratic Republic of the Congo and its neighbors affected by the epidemic. China has also sent public health experts and is maintaining a close collaboration with the World Health Organization and the African Union Commission.

In the context of foreign assistance and knowledge sharing, China often uses adjectives with connotations of openness and collaboration (e.g., shared, international, positive). In Excerpt 6, shared and international convey openness, while positive directly links cooperative outcomes with beneficial effects. Furthermore, depictions of medical personnel and grassroots communities are frequently accompanied by morally laudatory adjectives (e.g., heroic, heartfelt), as in Excerpt 7, where moral prestige is ascribed to actors, portraying them as ethical exemplars within the crisis narrative. Overall, China’s predication strategies construct an image of an “efficient, reliable, and willing-to-share” global public health partner.

*Excerpt* 6: We have done our best to provide anti-COVID-19 supplies and shared our practices in COVID-19 response. China was among the first to promise to make COVID-19 vaccines a global public good and to support waiving intellectual property rights on the vaccines.

*Excerpt* 7: In the first half of the year, the people of China put up a heroic fight against SARS and won a resounding victory.

By contrast, while the United States usage of adjectives in similar contexts also tends toward positive evaluation, the emphasis more often lies in underscoring national resources and strengths, frequently coupled with a sovereignty-first orientation. Common adjectives include many, military, central, and immediate, often used to describe military assistance, federal emergency measures, and domestic resource mobilization—thus constructing a national image of “overwhelming resources and execution capacity” and linking crisis management directly to national security. For Excerpt 8, “military, immediate, and central” highlight the speed, core status, and authority of resource deployment.

*Excerpt* 8: We need to act now to get shots in arms as fast as possible and expand access to oxygen, tests and treatments to save lives around the world. For the future, we need to create a new mechanism to finance global health security that builds on our existing development assistance, and a global health-threat council that is armed with the tools we need to monitor and identify emerging pandemics so that we can take immediate action.

Other adjectives, such as exceptional and enduring, underscore the United States’ long-term advantages in research and technology, perpetuating the “American exceptionalism” narrative. Meanwhile, adjectives like last and national frequently occur in policy contexts related to border closures and vaccine prioritization, pointing to sovereignty-first and domestic interest–centered principles, thereby reflecting a distinctly self-focused stance.

In sum, China consistently employs positive evaluations and cooperative language across crises to reinforce its identity as a “responsible global participant,” while the United States adjusts its predication strategies in line with the nature of the crisis and foreign policy needs, emphasizing national strength and sovereignty-first priorities. This divergence reflects each country’s strategic orientation when shaping its national image in multilateral settings, setting the stage for the next section’s analysis of how argumentation strategies rationalize identity construction and consolidate discursive legitimacy.

#### 4.2.3 Argumentation strategies

While naming and predication strategies enable positive self-representation and negative other-representation, argumentation strategies are essential for legitimizing these portrayals (72). In practice, these strategies employ topoi and fallacies, with topoi functioning as argumentative warrants that justify positive or negative attributions within an argumentative scheme. In the context of public health crises, Chinese and US leaders deploy topoi to rationalize favorable self-identities and unfavorable other-identities in their speeches. As shown in Table 6, both corpora rely on topoi of usefulness, responsibility, justice, danger, and threat to construct positive self-identity and negative identity of the “other.

**Table 6:** Topos used to construct a positive “self.”

Crisis	State	Topos
SARS	China	Responsibility, Usefulness, Fairness
H1N1	China	Responsibility, Usefulness, Fairness
	The United States	Responsibility, Usefulness
EBOLA	China	Responsibility, Usefulness, Fairness
	The United States	Responsibility, Usefulness
AIDS	China	Responsibility, Usefulness, Fairness
	The United States	Responsibility, Usefulness
COVIDS-19	China	Responsibility, Usefulness, Fairness, History
	The United States	Responsibility, Usefulness

In the positive self-identity dimension of argumentation strategies, China has predominantly employed the topos of usefulness, the topos of justice, and the topos of responsibility across various public health crises to reinforce a favorable national image. The topos of usefulness emphasizes the tangible outcomes of epidemic prevention and control measures; the topos of justice foregrounds the fairness of resource allocation, with particular attention to the needs of developing countries; and the topos of responsibility highlights China’s role as a major power committed to global welfare. For Excerpt, Excerpt 9 underscores the achievement of “bringing the epidemic under control and restoring normal life” (topos of usefulness); Excerpt 10 stresses the positioning of vaccines as a global public good, ensuring accessibility for developing countries (topos of justice); and Excerpt 11 juxtaposes historical contributions with current actions to signal China’s enduring sense of responsibility in safeguarding global public health security (topos of responsibility). Moreover, China has repeatedly reaffirmed its advocacy for intellectual property waivers on vaccines and large-scale international assistance (Excerpt 12), further consolidating its identity as a “responsible provider of global public goods.”

*Excerpt* 9: Since the start of this year, we, the 1.4 billion Chinese, undaunted by the strike of COVID-19, and with the government and the people united as one, have made all- out efforts to control the virus and speedily restore life and economy to normalcy.

*Excerpt* 10: Vaccination is our powerful weapon against COVID-19. I have stressed on many occasions the need to make vaccines a global public good and ensure vaccine accessibility and affordability in developing countries. Of pressing priority is to ensure the fair and equitable distribution of vaccines globally.

*Excerpt* 11: Seventy-five years ago, China made historic contributions to winning the World Anti-Fascist War and supported the founding of the United Nations. Today, with the same sense of responsibility, China is actively involved in the international fight against COVID-19, contributing its share to upholding global public health security.

*Excerpt* 12: China was among the first to promise to make COVID-19 vaccines a global public good and to support waiving intellectual property rights on the vaccines. China has provided over 2.2 billion doses of vaccines to more than 120 countries and international organizations.

While the United States likewise draws on the topos of usefulness and the topos of responsibility, its strategic focus differs. US discourse more explicitly accentuates its leadership position and irreplaceable role within the global public health system. This is often achieved by showcasing advances in medical technology, financial contributions, and emergency management capacity. For instance, Excerpt 1 highlights the mass production of ventilators for global distribution alongside progress in treatment technologies (topos of usefulness); Excerpt 13 underscores substantial financial commitments to combating HIV/AIDS, tuberculosis, and malaria, thereby signaling long-term dedication (topos of responsibility); and Excerpt 14 intertwines the advantages of the US economic system with global public health governance—first demonstrating capability and contributions, then asserting moral responsibility—thereby framing US leadership as both necessary and legitimate. These topoi have been recurrent across crises such as HIV/AIDS, H1N1, Ebola, and COVID-19, forming a durable discursive pattern.

*Excerpt* 13: We rapidly produced a record supply of ventilators—creating a surplus that allowed us to share them with friends and partners all around the globe. We pioneered life-saving treatments, reducing our fatality rate 85 per cent since April.

*Excerpt* 14: We have set aside $63 billion to carry forward the fight against HIV/AIDS, to end deaths from tuberculosis and malaria, to eradicate polio and to strengthen public health systems.

*Excerpt* 15: I believe that capitalism has been the greatest creator of wealth and opportunity that the world has ever known. We can roll back preventable disease and end the scourge of HIV/AIDS. We can stamp out pandemics that recognize no borders. That work may not be on television right now, but as we demonstrated in reversing the spread of Ebola, it can save more lives than anything else we can do.

In constructing the identity of the “other,” China tends to avoid direct negative accusations. Instead, it foregrounds strengthening global cooperation, adhering to scientific guidance, and rejecting political stigmatization. Through this discourse, Chinese leaders seek to reinforce China’s image as a responsible major power, highlighting cooperation with international organizations and other countries to address shared global challenges (see Excerpt 16). In multilateral settings, constructing a “negative Other” would undermine China’s cooperative image and contradict its vision of a Global Community of Health for Humankind.

*Excerpt* 16: We should follow the guidance of science, give full play to the leading role of the World Health Organization, and launch a joint international response to beat this pandemic. Any attempt of politicizing the issue or stigmatization must be rejected.

*Excerpt* 17: China is the largest developing country in the world, a country that is committed to peaceful, open, cooperative and common development. We will never seek hegemony, expansion, or sphere of influence.

As evident in the above excerpts, China’s discourse of “cooperation” and “development” also carries an anti-hegemonic dimension. By advocating multilateralism and opposing unilateralism, China implicitly questions and weakens the legitimacy of the US-led liberal international order, while promoting an alternative vision of a more equal and inclusive international order.

In contrast, during the initial outbreak of COVID-19, the United States frequently employed the topos of threat, the topos of responsibility, and the topos of humiliation to construct a distinctly negative “other,” (see Table 7), portraying China as the origin of the pandemic and accusing it alongside the World Health Organization of misleading the international community regarding critical epidemic information (Excerpts 17–19). Such strategies not only reinforced the geopolitical antagonism between China and the United States but also reflected domestic nationalist sentiment and a strategic competition mindset within the US political discourse.

**Table 7:** Topos used to construct a negative “other” in US speech during COVID-19.

Topos	Cliams
Threat	Regard the coronavirus as the “China virus”
Shame	Accuse China and the WHO of spreading “false” information
Responsibility	It is demanded that China be held accountable

*Excerpt* 18: We have waged a fierce battle against the invisible enemy—the China Virus — which has claimed countless lives in 188 countries.

*Excerpt* 19: The Chinese government, and the World Health Organization—which is virtually controlled by China—falsely declared that there was no evidence of human- to-human transmission.

*Excerpt* 20: As we pursue this bright future, we must hold accountable the nation which unleashed this plague onto the world: China.

Overall, China’s argumentation strategies center on fostering a positive self-identity and promoting global cooperation, whereas the United States, while also emphasizing a positive self-identity, more frequently engages in constructing a negative “other” as a means of legitimizing its leadership role. This divergence underscores the fundamental differences in the two countries’ foreign policy objectives and value orientations, offering important insight into the international impact of their crisis discourses.

## 5 Discussion and implication

This study employs a combination of discourse-historical analysis (DHA) and corpus-based methods to examine speeches delivered by Chinese and American leaders at the United Nations General Assembly (UNGA) between 2003 and 2023 in the context of global public health crises. The findings reveal that national identity is dynamically constructed in times of crisis. Over the past two decades, China’s discourse has shown strong continuity and stability, consistently emphasizing the themes of “cooperation–development–multilateralism,” which have reinforced its image as a “responsible major power.” The stable nature of China’s political system and its long-term strategic orientation have ensured a high degree of coherence and consistency in diplomatic discourse, with leadership changes not leading to fundamental policy shifts. Chinese diplomatic rhetoric has continually centered on key concepts such as “peaceful development,” “win-win cooperation,” and “a community with a shared future for mankind.” By maintaining narrative continuity, China has gradually shaped its national identity and accumulated discursive power on the international stage. This stable discursive orientation has been particularly evident during global health crises such as the COVID-19 pandemic, demonstrating a cooperative stance and offering a communicative framework for international governance.

In contrast, US discourse has shown greater fluctuation, oscillating between “universal values” and “national interests,” as well as between “leadership responsibility” and “defensive posture.” Such variation stems partly from partisan differences, as different administrations adjust their diplomatic rhetoric and policy priorities, and partly from structural factors, as a leading power in the international order, the United States adapts its diplomatic narrative according to changes in global power structures and the nature of crises. However, regardless of party turnover or strategic adjustment, its core narrative remains focused on “security, freedom, and leadership,” aimed at maintaining the US-led international order and its discursive dominance. This pattern of apparent change but underlying continuity reveals the hegemonic nature of US diplomatic discourse, constantly reaffirming its leadership position and institutional legitimacy across crisis contexts, reproducing its dominance through language. However, such rhetorical inconsistency may challenge the stability of global health governance, as fluctuating narratives can hinder mutual trust and consensus among international partners.

These differences supports a central proposition of national identity theory: identity is socially constructed through interaction between the self and the other (4), and crises intensify this interaction’s conflictual and dynamic nature. Building on this, the study proposes the concept of “crisis-driven short-term identity adjustment,” suggesting that crises are not only about health and security but also serve as opportunities for states to redefine their international roles.

Theoretically, this study extends the application of national identity theory to crisis contexts, demonstrating that identity construction is situational, dynamic, and malleable, yet simultaneously shaped by institutional structures and role expectations in the international system. Practically, the findings shed light on the divergent identity logics guiding China and the United States in global governance and offer insights into their potential pathways of cooperation and competition in future public health crises. Moreover, the findings have important policy implications for international organizations and global health governance. Discourse analysis reveals how member states reshape their image and position during negotiations, offering insights for the World Health Organization’s (WHO) pandemic treaty talks and other multilateral health negotiations. Understanding the narrative logic of national identities can help identify common ground, reduce discursive confrontation, and enhance the sustainability of international public health cooperation.

This study has several limitations. First, the data sources are limited to English versions of speeches by Chinese and US leaders in the UN General Assembly’s general debates. The research provides a deep but narrow perspective on discourse strategies. Therefore, future research could expand to other multilateral diplomatic settings, such as the G20 or the World Health Assembly, to improve representativeness. Second, the analysis mainly focuses on elite political discourse, the analytical scope of this study remains relatively limited. Future research may integrate media coverage and public opinion, it could help explore how identity construction is disseminated and recognized at the societal level. Finally, this study combines quantitative and qualitative analyses to minimize interpretative bias, while qualitative interpretation remains context-dependent, reflecting the distinct policy orientations and institutional discourses of the two countries. Future research could integrate corpus-based quantitative modeling with expert interviews to enhance interpretative validity and further reduce context-specific constraints. Besides, integrating perspectives from international relations, public health, and communication studies will further illuminate how crisis narratives intersect with power structures and institutional arrangements, contributing to a more comprehensive framework for analyzing global crisis governance.

## Data Availability

The original contributions presented in the study are included in the article/supplementary material, further inquiries can be directed to the corresponding author.
